# The problem-solving method: Efficacy for learning and motivation in the field of physical education

**DOI:** 10.3389/fpsyg.2022.1041252

**Published:** 2023-01-25

**Authors:** Ghaith Ezeddine, Nafaa Souissi, Liwa Masmoudi, Khaled Trabelsi, Luca Puce, Cain C. T. Clark, Nicola Luigi Bragazzi, Maher Mrayah

**Affiliations:** ^1^High Institute of Sport and Physical Education of Sfax, University of Sfax, Sfax, Tunisia; ^2^Research Unit of the National Sports Observatory (ONS), Tunis, Tunisia; ^3^Research Laboratory: Education, Motricity, Sport and Health, EM2S, LR19JS01, University of Sfax, Sfax, Tunisia; ^4^Department of Neuroscience, Rehabilitation, Ophthalmology, Genetics, Maternal and Child Health (DINOGMI), University of Genoa, Genoa, Italy; ^5^Centre for Intelligent Healthcare, Coventry University, Coventry, United Kingdom; ^6^Laboratory for Industrial and Applied Mathematics, Department of Mathematics and Statistics, York University, Toronto, ON, Canada; ^7^High Institute of Sport and Physical Education of Ksar Saîd, University Manouba, UMA, Manouba, Tunisia

**Keywords:** problem-solving method, traditional method, motivation, learning, students

## Abstract

**Background:**

In pursuit of quality teaching and learning, teachers seek the best method to provide their students with a positive educational atmosphere and the most appropriate learning conditions.

**Objectives:**

The purpose of this study is to compare the effects of the problem-solving method vs. the traditional method on motivation and learning during physical education courses.

**Methods:**

Fifty-three students (*M*^age^ 15 ± 0.1 years), in their 1st year of the Tunisian secondary education system, voluntarily participated in this study, and randomly assigned to a control or experimental group. Participants in the control group were taught using the traditional methods, whereas participants in the experimental group were taught using the problem-solving method. Both groups took part in a 10-hour experiment over 5 weeks. To measure students' situational motivation, a questionnaire was used to evaluate intrinsic motivation, identified regulation, external regulation, and amotivation during the first (T0) and the last sessions (T2). Additionally, the degree of students' learning was determined *via* video analyses, recorded at T0, the fifth (T1), and T2.

**Results:**

Motivational dimensions, including identified regulation and intrinsic motivation, were significantly greater (all *p* < 0.001) in the experimental vs. the control group. The students' motor engagement in learning situations, during which the learner, despite a degree of difficulty performs the motor activity with sufficient success, increased only in the experimental group (*p* < 0.001). The waiting time in the experimental group decreased significantly at T1 and T2 vs. T0 (all *p* < 0.001), with lower values recorded in the experimental vs. the control group at the three-time points (all *p* < 0.001).

**Conclusions:**

The problem-solving method is an efficient strategy for motor skills and performance enhancement, as well as motivation development during physical education courses.

## 1. Introduction

The education of children is a sensitive and poignant subject, where the wellbeing of the child in the school environment is a key issue (Ergül and Kargin, 2014). For this, numerous research has sought to find solutions to the problems of the traditional method, which focuses on the teacher as an instructor, giver of knowledge, arbiter of truth, and ultimate evaluator of learning (Ergül and Kargin, 2014; Cunningham and Sood, 2018). From this perspective, a teachers' job is to present students with a designated body of knowledge in a predetermined order (Arvind and Kusum, 2017). For them, learners are seen as people with “knowledge gaps” that need to be filled with information. In this method, teaching is conceived as the act of transmitting knowledge from point A (responsible for the teacher) to point B (responsible for the students; Arvind and Kusum, 2017). According to Novak (2010), in the traditional method, the teacher is the one who provokes the learning.

The traditional method focuses on lecture-based teaching as the center of instruction, emphasizing delivery of program and concept (Johnson, 2010; Ilkiw et al., 2017; Dickinson et al., 2018). The student listens and takes notes, passively accepts and receives from the teacher undifferentiated and identical knowledge (Bi et al., 2019). Course content and delivery are considered most important, and learners acquire knowledge through exercise and practice (Johnson et al., 1998). In the traditional method, academic achievement is seen as the ability of students to demonstrate, replicate, or convey this designated body of knowledge to the teacher. It is based on a transmissive model, the teacher contenting themselves with exchanging and transmitting information to the learner. Here, only the “knowledge” and “teacher” poles of the pedagogical triangle are solicited. The teacher teaches the students, who play the role of the spectator. They receive information without participating in its creation (Perrenoud, 2003). For this, researchers invented a new student-centered method with effects on improving students' graphic interpretation skills and conceptual understanding of kinematic motion represent an area of contemporary interest (Tebabal and Kahssay, 2011). Indeed, in order to facilitate the process of knowledge transfer, teachers should use appropriate methods targeted to specific objectives of the school curricula.

For instance, it has been emphasized that the effectiveness of any educational process as a whole relies on the crucial role of using a well-designed pedagogical (teaching and/or learning) strategy (Kolesnikova, 2016).

Alternate to a traditional method of teaching, Ergül and Kargin (2014), proposed the problem-solving method, which represents one of the most common student-centered learning strategies. Indeed, this method allows students to participate in the learning environment, giving them the responsibility for their own acquisition of knowledge, as well as the opportunity for the understanding and structuring of diverse information.

For Cunningham and Sood (2018), the problem-solving method may be considered a fundamental tool for the acquisition of new knowledge, notably learning transfer. Moreover, the problem-solving method is purportedly efficient for the development of manual skills and experiential learning (Ergül and Kargin, 2014), as well as the optimization of thinking ability. Additionally, the problem-solving method allows learners to participate in the learning environment, while giving them responsibility for their learning and making them understand and structure the information (Pohan et al., 2020). In this context, Ali (2019) reported that, when faced with an obstacle, the student will have to invoke his/her knowledge and use his/her abilities to “break the deadlock.” He/she will therefore make the most of his/her potential, but also share and exchange with his/her colleagues (Ali, 2019). Throughout the process, the student will learn new concepts and skills. The role of the teacher is paramount at the beginning of the activity, since activities will be created based on problematic situations according to the subject and the program. However, on the day of the activity, it does not have the main role, and the teacher will guide learners in difficulty and will allow them to manage themselves most of the time (Ali, 2019).

The problem-solving method encourages group discussion and teamwork (Fidan and Tuncel, 2019). Additionally, in this pedagogical approach, the role of the teacher is a facilitator of learning, and they take on a much more interactive and less rebarbative role (Garrett, 2008).

For the teaching method to be effective, teaching should consist of an ongoing process of making desirable changes among learners using appropriate methods (Ayeni, 2011; Norboev, 2021). To bring about positive changes in students, the methods used by teachers should be the best for the subject to be taught (Adunola et al., 2012). Further, suggests that teaching methods work effectively, especially if they meet the needs of learners since each learner interprets and answers questions in a unique way. Improving problem-solving skills is a primary educational goal, as is the ability to use reasoning. To acquire this skill, students must solve problems to learn mathematics and problem-solving (Hu, 2010); this encourages the students to actively participate and contribute to the activities suggested by the teacher. Without sufficient motivation, learning goals can no longer be optimally achieved, although learners may have exceptional abilities. The method of teaching employed by the teachers is decisive to achieve motivational consequences in physical education students (Leo et al., 2022). Pérez-Jorge et al. (2021) posited that given we now live in a technological society in which children are used to receiving a large amount of stimuli, gaining and maintaining their attention and keeping them motivated at school becomes a challenge for teachers.

Fenouillet (2012) stated that academic motivation is linked to resources and methods that improve attention for school learning. Furthermore, Rolland (2009) and Bessa et al. (2021) reported a link between a learner's motivational dynamics and classroom activities. The models of learning situations, where the student is the main actor, directly refers to active teaching methods, and that there is a strong link between motivation and active teaching (Rossa et al., 2021). In the same context, previous reports assert that the motivation of students in physical education is an important factor since the intra-individual motivation toward this discipline is recognized as a major determinant of physical activity for students (Standage et al., 2012; Luo, 2019; Leo et al., 2022). Further, extensive research on the effectiveness of teaching methods shows that the quality of teaching often influences the performance of learners (Norboev, 2021). Ayeni (2011) reported that education is a process that allows students to make changes desirable to achieve specific results. Thus, the consistency of teaching methods with student needs and learning influences student achievement. This has led several researchers to explore the impact of different teaching strategies, ranging from traditional methods to active learning techniques that can be used such as the problem-solving method (Skinner, 1985; Darling-Hammond et al., 2020).

In the context of innovation, Blázquez (2016) emphasizes the importance of adopting active methods and implementing them as the main element promoting the development of skills, motivation and active participation. Pedagogical models are part of the active methods which, together with model-based practice, replace traditional teaching (Hastie and Casey, 2014; Casey et al., 2021). Thus, many studies have identified pedagogical models as the most effective way to place students at the center of the teaching-learning process (Metzler, 2017), making it possible to assess the impact of physical education on learning students (Casey, 2014; Rivera-Pérez et al., 2020; Manninen and Campbell, 2021). Since each model is designed to focus on a specific program objective, each model has limitations when implemented in isolation (Bunker and Thorpe, 1982; Rivera-Pérez et al., 2020). Therefore, focusing on developing students' social and emotional skills and capacities could help them avoid failure in physical education (Ang and Penney, 2013). Thus, the current emergence of new pedagogical models goes with their hybridization with different methods, which is a wave of combinations proposed today as an innovative pedagogical strategy. The incorporation of this type of method in the current education system is becoming increasingly important because it gives students a greater role, participation, autonomy and self-regulation, and above all it improves their motivation (Puigarnau et al., 2016). The teaching model of personal and social responsibility, for example, is closely related to the sports education model because both share certain approaches to responsibility (Siedentop et al., 2011). One of the first studies to use these two models together was Rugby (Gordon and Doyle, 2015), which found significant improvements in student behavior. Also, the recent study by Menendez and Fernandez-Rio (2017) on educational kickboxing.

Previous studies have indicated that hybridization can increase play, problem solving performance and motor skills (Menendez and Fernandez-Rio, 2017; Ward et al., 2021) and generate positive psychosocial consequences, such as pleasure, intention to be physically active and responsibility (Dyson and Grineski, 2001; Menendez and Fernandez-Rio, 2017).

But despite all these research results, the picture remains unclear, and it remains unknown which method is more effective in improving students' learning and motivation. Given the lack of published evidence on this topic, the aim of this study was to compare the effects of problem-solving vs. the traditional method on students' motivation and learning.

We hypothesized would that the problem-solving method would be more effective in improving students' motivation and learning better than the traditional method.

## 2. Materials and method

### 2.1. Participants

Fifty-three students, aged 15–16 (*M*^age^ 15 ± 0.1 years), in their 1st year of the Tunisian secondary education system, voluntarily participated in this study. All participants were randomly chosen. Repeating students, those who practice handball activity in civil/competitive/amateur clubs or in the high school sports association, and students who were absent, even for one session, were excluded. The first class consisted of 30 students (16 boys and 14 girls), who represented the experimental group and followed basic courses on a learning method by solving problems. The second class consisted of 23 students (10 boys and 13 girls), who represented the control group and followed the traditional teaching method. The total duration was spread over 5 weeks, or two sessions per week and each session lasted 50 min.

University research ethics board approval (CPPSUD: 0295/2021) was obtained before recruiting participants who were subsequently informed of the nature, objective, methodology, and constraints. Teacher, school director, parental/guardian, and child informed consent was obtained prior to participation in the study.

### 2.2. Procedure

Before the start of the experiment, the participants were familiarized with the equipment and the experimental protocol in order to ensure a good learning climate. For this and to mitigate the impact of the observer and the cameras on the students, the two researchers were involved prior to the data collection in a week of familiarization by making test recordings with the classes concerned.

An approach of a teaching cycle consisting of 10 sessions spread over 5 weeks, amounting to two sessions per week. Physical education classes were held in the morning from 8 a.m. to 9 a.m., with a single goal for each session that lasted 50 min. The cyclic programs were produced by the teacher responsible for carrying out the experiment with 18 years of service. To do this, the students had the same lessons with the same objectives, only pedagogy that differs: the experimental group worked using problem-solving pedagogy, while the control group was confronted with traditional pedagogy. The sessions took place in a handball field 40 m long and 20 m wide. Examples of training sessions using the problem-solving pedagogy and the traditional pedagogy are presented in Table 1. In addition, a motivation questionnaire, the Situational Motivation Scale (SIMS; Guay et al., 2000), was administered to learners at the end of the session (i.e., in the beginning, and end of the cycle). Each student answered the questions alone and according to their own ideas. This questionnaire was taken in a classroom to prevent students from acting abnormally during the study. It lasted for a maximum of 10 min.

**Table 1:** Example of activities for the different sessions.

**Problem-solving method**	**Traditional method**
**T0**	**T0**
Evaluation test at the beginning of the cycle	Evaluation of the beginning of the cycle
The class will be divided into two groups: the first group will play a game of dodgeball. The second will perform the test through a two-man ball climb to the halfway line and then return to the goal by dribbling to attempt a shot. Then, learners change roles. The first group will do the test and the second group will play dodgeball.	The court is divided into three parts across the width. The students in turn are divided into teams of three and will play 3#3 on a third of the field. The winners will play among themselves.
**T1**	**T1**
**Situation 1**	**Situation 1**
The court is divided into three parts along the length. The students carry out a two-man ball lift in the presence of an active defender.	The work is done along the length of the court. The students perform a three-way ball run.
**Situation 2**	**Situation 2**
The court is divided into two parts along the length. The students carry out a three-way ball attack in the presence of two active defenders.	The same exercise, but students play crisscross.
**Situation 3**	**Situation 3**
Progress toward the opposing camp in crisscross and attempt a shot.	The work is done on a half court, students are divided in three groups on the 9 m line, they passes a ball and follows to finally shoot toward the cage.
**Situation 4**	
The students perform a four-man high ball in the presence of three active defenders.	
**T2**	**T2**
**Evaluation of the end of the cycle**	**Evaluation of the end of the cycle**
Divide the students into two groups: the first group is in turn divided into two teams, each starting from the sideline. At the signal, the student starts to dribble, goes around a cone and moves toward the cage to attempt a shot. The second group is divided into four teams to play 4#4 on a quarter of the court. Then, they will switch roles.	**Situation 1** The work is done on half court. These students who are going to do a two-man ball climb then one will dribble in slalom to shoot toward the cage and the second will become a goalkeeper then they will change roles.
	**Situation 2**
	The students perform a half match of 4#4.

Two diametrically opposed cameras were installed so to film all the movements and behaviors of each student and teacher during the three sessions [(i) test at the start of the cycle (T0), (ii) in the middle of the cycle (T1), and (iii) test at the end of the cycle (T2)]. These sessions had the same content and each consisted of four phases: the getting started, the warm-up, the work up (which consisted of three situations: first, the work was goes up the ball to two to score in the goal following a shot. Second, the same principle as the previous situation but in the presence of a defender. Finally, third, a match 7 ≠ 7), and the cooling down These recordings were analyzed using a Learning Time Analysis System grid (LTAS; Brunelle et al., 1988). This made it possible to measure individual learning by coding observable variables of the behavior of learners in a learning situation.

### 2.3. Data collection and analysis

#### 2.3.1. The motivation questionnaire

In this study, in order to measure the situational motivation of students, the situational motivation scale (SIMS; Guay et al., 2000), which used. This questionnaire assesses intrinsic motivation, identified regulation, external regulation and amotivation. SIMS has demonstrated good reliability and factor validity in the context of physical education in adolescents (Lonsdale et al., 2011). The participants received exact instructions from the researchers in accordance with written instructions on how to conduct the data collection. Participants completed the SIMS anonymously at the start of a physical education class. All students had the opportunity to write down their answers without being observed and to ask questions if anything was unclear. To minimize the tendency to give socially desirable answers, they were asked to answer as honestly as possible, with the confidence that the teacher would not be able to read their answers and that their grades would not be affected by how they responded. The SIMS questionnaire was filled at T0 and T2. This scale is made up of 16 items divided into four dimensions: intrinsic motivation, identified regulation, external regulation and amotivation. Each item is rated on a 7-point Likert scale ranging from 1 (which is the weakest factor) “not at all” to 7 (which is the strongest factor) “exactly matches.”

In order to assess the internal consistency of the scales, a Cronbach alpha test was conducted (Cronbach, 1951). The internal consistency of the scales was acceptable with reliability coefficients ranging from 0.719 to 0.87. The coefficient of reliability was 0.8.
In the present study, Cronbach's alphas were: intrinsic motivation = 0.790; regulation identified = 0.870; external regulation = 0.749; and amotivation = 0.719.

#### 2.3.2. Camcorders

The audio-visual data collection was conducted using two Sony camcorders (Model; Handcam 4K) with a wireless microphone with a DJ transmitter-receiver (VHF 10HL F4 Micro HF) with a range of 80 m (Maddeh et al., 2020). The collection took place over a period of 5 weeks, with three captures for each class (three sessions of 50 min for each at T0, T1, and T2). Two researchers were trained in the procedures and video capture techniques. The cameras were positioned diagonally, in order to film all the behavior of the students and teacher on the set.

#### 2.3.3. The Learning Time Analysis System (LTAS)

To measure the degree of student learning, the analysis of videos recorded using the LTAS grid by Brunelle et al. (1988) was used, at T0, T1, and T2. This observation system with predetermined categories uses the technique of observation by small intervals (i.e., 6 s) and allows to measure individual learning by coding observable variables of their behaviors when they have been in a learning situation. This grid also permits the specification of the quantity and quality with which the participants engaged in the requested work and was graded, broadly, on two characteristics: the type of situation offered to the group by the teacher and the behavior of the target participant. The situation offered to the group was subdivided into three parts: preparatory situations; knowledge development situations, and motor development situations.

The observations and coding of behaviors are carried out “at intervals.” This technique is used extensively in research on behavior analysis. The coder observes the teaching situation and a particular student during each interval (Brunelle et al., 1988). It then makes a decision concerning the characteristic of the observed behavior. The 6-s observation interval is followed by a coding interval of 6 s too. A cassette tape recorder is used to regulate the observation and recording intervals. It is recorded for this purpose with the indices “observe” and “code” at the start of each 6-s period. During each coding unit, the observer answered the following questions: What is the type of situation in which the class group finds itself? If the class group is in a learning situation proper, in what form of commitment does the observed student find himself? The abbreviations representing the various categories of behavior have been entered in the spaces which correspond to them. The coder was asked to enter a hyphen instead of the abbreviation when the same categories of behavior follow one another in consecutive intervals (Brunelle et al., 1988).

During the preparatory period, the following behaviors were identified and analyzed:

- Deviant behavior: The student adopts a behavior incompatible with a listening attitude or with the smooth running of the preparatory situations.
- Waiting time: The student is waiting without listening or observing.
- Organized during: The student is involved in a complementary activity that does not represent a contribution to learning (e.g., regaining his place in a line, fetching a ball that has just left the field, replacing a piece of equipment).

During the motor development situations, the following behaviors were identified and analyzed:

- Motor engagement 1: The participant performs the motor activity with such easy that it can be inferred that their actions have little chance to engage in a learning process.
- Motor engagement 2: The participant-despite a certain degree of difficulty, performs the motor activity with sufficient success, which makes it possible to infer that they are in the process of learning.
- Motor engagement 3: The participant performs the motor activity with such difficulty that their efforts have very little chance of being part of a learning process.

### 2.4. Statistical analysis

Statistical tests were performed using statistical software 26.0 for windows (SPSS, Inc, Chicago, IL, USA). Data are presented in text and tables as means ± standard deviations and in figures as means and standard errors. Once the normal distribution of data was confirmed by the Shapiro-Wilk *W*-test, parametric tests were performed. Analysis of the results was performed using a mixed 2-way analysis of variance (ANOVA): Groups × Time with repeated measures.

For the learning parameters, the ANOVA took the following form: 2 Groups (Control Group vs. Experimental Group) × 3 Times (T0, T1, and T2).
For the dimensions of motivation, the ANOVA took the following form: 2 Groups (Control Group vs. Experimental Group) × 2 Time (T0 vs. T2).

In instances where the ANOVA showed a significant effect, a Bonferroni *post-hoc* test was applied in order to compare the experimental data in pairs, otherwise by an independent or paired Student's *T*-test. Effect sizes were calculated as partial eta-squared η*p*^2^ to estimate the meaningfulness of significant findings, where η*p*^2^ values of 0.01, 0.06, and 0.13 represent small, moderate, and large effect sizes, respectively (Lakens, 2013). All observed differences were considered statistically significant for a probability threshold lower than *p* < 0.05.

## 3. Results

Table 2 shows the results of learning variables during the preparatory and the development learning periods at T0, T1, and T2, in the control group and the experimental group.

**Table 2:** Comparison of learning variables using two teaching methods in physical education.

**Variables**		**Groups**	**Means** ±**SD**			**Groups effect**			**Learning effect**			**Groups** × **learning interaction**		

			**T0**	**T1**	**T2**	*F*_(1, 51)_	***p*** **-value**	$\eta_{p}^{2}$	*F*_(2, 102)_	***p*** **-value**	$\eta_{p}^{2}$	*F*_(2, 102)_	***p*** **-value**	$\eta_{p}^{2}$
Preparatory period	Deviant behavior	Control group	40.7 ± 15	38.9 ± 11	30.3 ± 11.5^#$^	90.524	0.000	0.640	61.332	0.000	0.546	5.070	0.008	0.090
		Experimental group	26.1 ± 6.2*	19.3 ± 5.7^*#^	7.2 ± 3.4^*#$^									
	Appropriate engagement	Control group	68 ± 10.9	64.3 ± 10	57.5 ± 5.4^#$^	0.661	0.420	0.013	4.219	0.017	0.076	62.812	0.000	0.552
		Experimental group	56.5 ± 3.3*	64 ± 2.4^#^	65.9 ± 1.7^*#^									
	Waiting time	Control group	82.9 ± 2.9	87.9 ± 3^#^	97 ± 3.5^#$^	2,902.065	0.000	0.983	56.068	0.000	0.524	683.062	0.000	0.931
		Experimental group	70.6 ± 2.9*	67.3 ± 3.1^*#^	47.8 ± 1.4^*#$^									
Motor development	Motor engagement 2	Control group	14.2 ± 25.7	20.9 ± 19	61.1 ± 33.8^#$^	34.126	0.000	0.401	80.626	0.000	0.613	8.553	0.000	0.144
		Experimental group	38.4 ± 51.7	55.3 ± 42.6*	131.8 ± 28.6^*#$^									
	Motor engagement 3	Control group	45.9 ± 25.4	40.2 ± 18.9	18 ± 31.8^#^	1.683	0.200	0.032	31.219	0.000	0.380	3.984	0.022	0.072
		Experimental group	68.9 ± 51.3	54.1 ± 41.5	9.3 ± 27.9^#$^									
	Organized during	Control group	13.1 ± 2.3	12.5 ± 1.3	11 ± 4.2^#^	29.983	0.000	0.370	16.687	0.000	0.247	1.075	0.345	0.021
		Experimental group	14.6 ± 1.1	15 ± 0.6*	12.9 ± 0.4^*#$^									

^*^Significantly different from control group at p <0.05.

^#^Significantly different from T0 at p <0.05.

^$^Significantly different from T1 at p <0.05.

For motor engagement 1 (ME1), the time devoted to this variable is equal zero for the three measurement times (T0, T1, and T2).

The analysis of variance of two factors with repeated measures showed a significant effect of group, learning, and group learning interaction for the deviant behavior. The *post-hoc* test revealed significantly less frequent deviant behaviors in the experimental than in the control group at T0, T1, and T2 (all *p* < 0.001). Additionally, the deviant behavior decreased significantly at T1 and T2 compared to T0 for both groups (all *p* < 0.001).

For appropriate engagement, there were no significant group effect, a significant learning effect, and a significant group learning interaction effect. The *post-hoc* test revealed that compared to T0, Appropriate engagement recorded at T1 and T2 increased significantly (*p* = 0.032; *p* = 0.031, respectively) in the experimental group, whilst it decreased significantly in the control group (*p* < 0.001). Additionally, Appropriate engagement was higher in the experimental vs. control group at T1 and T2 (all *p* < 0.001).

For waiting time, a significant interaction in terms of group effect, learning, and group learning was found. The *post-hoc* test revealed that waiting time was higher at T1 and T2 vs. T0 (all *p* < 0.001) in the control group. In addition, waiting time in the experimental group decreased significantly at T1 and T2 vs. T0 (all *p* < 0.001), with higher values recorded at T2 vs. T1 (*p* = 0.025). Additionally, lower values were recorded in the experimental group vs. the control group at the three-time points (all *p* < 0.001).

For Motor engagement 2, a significant group, learning, and group-learning interaction effect was noted. The *post-hoc* test revealed that Motor engagement 2 increased significantly in both groups at T1 (*p* < 0.0001) and T2 (*p* < 0.0001) vs. T0 (*p* = 0.045), with significantly higher values recorded in the experimental group at T1 and T2.

Regarding Motor engagement 3, a non-significant group effect was reported. Contrariwise, a significant learning effect and group learning interaction was reported (Table 1). The *post-hoc* test revealed a significant decrease in the control group and the experimental group at T1 (*p* = 0.294) at T2 (*p* = 0.294) vs. T0 (*p* = 0.0543). In addition, a non-significant difference between the two groups was found.

A significant group and learning effect was noted for the organized during, and a non-significant group learning interaction. For organized during, the paired Student *T*-test showed a significant decrease in the control group and the experimental group (all *p* < 0.001). The independent Student *T*-test revealed a non-significant difference between groups at the three-time points.

Results of the motivational dimensions in the control group and the experimental group recorded at T0 and T2 are presented in Table 3.

**Table 3:** Comparison of the four motivational dimensions in two teaching methods in physical education.

**Dimensions**		**Means** ±**SD**		**Group effect**			**Learning effect**			**Group** × **learning interaction**		

		**T0**	**T2**	*F*_(1, 51)_	***p*** **-value**	$\eta_{p}^{2}$	*F*_(2, 102)_	***p*** **-value**	$\eta_{p}^{2}$	*F*_(2, 102)_	***p*** **-value**	$\eta_{p}^{2}$
Intrinsic motivation	Control group	4.4 ± 2.1	3.3 ± 1.3^#^	35.859	<0.001	0.413	0.692	0.409	0.013	17.206	<0.001	0.252
	Experimental group	5.5 ± 1.4*	6.2 ± 0.8^*#^									
Identified regulation	Control group	4.2 ± 1.8	4.4 ± 1.1	17.682	<0.001	0.257	1.341	0.252	0.026	0.236	0.629	0.005
	Experimental group	5.4 ± 1.5*	5.8 ± 1.2^*#^									
External regulation	Control group	4.3 ± 1.4	4.2 ± 1	11.892	0.001	0.189	3.726	0.059	0.068	1.821	0.183	0.034
	Experimental group	3.7 ± 1.1	3 ± 1.2^#*^									
Amotivation	Control group	3.5 ± 1.3	3.9 ± 1.1	7.828	0.007	0.133	0.023	0.881	0.000	3.145	0.082	0.058
	Experimental group	3.2 ± 1.1	2.9 ± 1.1^#*^									

^*^Significantly different from control group at p <0.05.

^#^Significantly different from T0 at p <0.05.

For intrinsic motivation, a significant group effect and group learning interaction and also a non-significant learning effect was found. The *post-hoc* test indicated that the intrinsic motivation decreased significantly in the control group (*p* = 0.029), whilst it increased in the experimental group (*p* = 0.04). Additionally, the intrinsic motivation of the experimental group was higher at T0 (*p* = 0.026) and T2 (*p* < 0.001) compared to that of the control group.

For the identified regulation, a significant group effect, a non-significant learning effect and group learning interaction were reported. The paired Student's *T*-test revealed that from T0 to T1, the identified motivation increased significantly only in the experimental group (*p* = 0.022), while it remained unchanged in the control group. The independent Student's *T*-test revealed that the identified regulation recorded in the experimental group at T0 (*p* = 0.012) and T2 (*p* < 0.001) was higher compared to that of the control group.

The external regulation presents a significant group effect. In addition, a non-significant learning effect and group learning interaction were reported. The paired Student's *T*-test showed that the external regulation decreased significantly in the experimental group (*p* = 0.038), whereas it remained unchanged in the control group. Further, the independent Student's *T*-test revealed that the external regulation recorded at T2 was higher in the control group vs. the experimental group (*p* < 0.001).

Relating to amotivation, results showed a significant group effect. Furthermore, a non-significant learning effect and group learning interaction were reported. The paired Student's *T*-test showed that, from T0 to T2, amotivation decreased significantly in the experimental group (*p* = 0.011) and did not change in the control group. The independent Student *T*-test revealed that amotivation recorded at T2 was lower in the experimental compared to the control group (*p* = 0.002).

## 4. Discussion

The main purpose of this study was to compare the effects of the problem-solving vs. traditional method on motivation and learning during physical education courses. The results revealed that the problem-solving method is more effective than the traditional method in increasing students' motivation and improving their learning. Moreover, the results showed that mean wait times and deviant behaviors decreased using the problem-solving method. Interestingly, the average time spent on appropriate engagement increased using the problem-solving method compared to the traditional method. When using the traditional method, the average wait times increased and, as a result, the time spent on appropriate engagement decreased. Then, following the decrease in deviant behaviors and waiting times, an increase in the time spent warming up was evident (i.e., appropriate engagement). Indeed, there was an improvement in engagement time using the problem-solving method and a decrease using the traditional method. On the other hand, there was a decrease in motor engagement 3 in favor of motor engagement 2. Indeed, it has been shown that the problem-solving method has been used in the learning process and allows for its improvement (Docktor et al., 2015). In addition, it could also produce better quality solutions and has higher scores on conceptual and problem-solving measures. It is also a good method for the learning process to enhance students' academic performance (Docktor et al., 2015; Ali, 2019). In contrast, the traditional method limits the ability of teachers to reach and engage all students (Cook and Artino, 2016). Furthermore, it produces passive learning with an understanding of basic knowledge which is characterized by its weakness (Goldstein, 2016). Taken together, it appears that the problem-solving method promotes and improves learning more than the traditional method.

It should be acknowledged that other factors, such as motivation, could influence learning. In this context, our results showed that the method of problem-solving could improve the motivation of the learners. This motivation includes several variables that change depending on the situation, namely the intrinsic motivation that pushes the learner to engage in an activity for the interest and pleasure linked to the practice of the latter (Komarraju et al., 2009; Guiffrida et al., 2013; Chedru, 2015). The student, therefore, likes to learn through problem-solving and neglects that of the traditional method. These results are concordant with others (Deci and Ryan, 1985; Chedru, 2015; Ryan and Deci, 2020). Regarding the three forms of extrinsic motivation: first, extrinsic motivation by an identified regulation which manifests itself in a high degree of self-determination where the learner engages in the activity because it is important for him (Deci and Ryan, 1985; Chedru, 2015). This explains the significant difference between the two groups. Then, the motivation by external regulation which is characterized by a low degree of self-determination such as the behavior of the learner is manipulated by external circumstances such as obtaining rewards or the removal of sanctions (Deci and Ryan, 1985; Chedru, 2015). For this, the means of this variable decreased for the experimental group which is intrinsically motivated. He does not need any reward to work and is not afraid of punishment because he is self-confident. Third, amotivation is at the opposite end of the self-determination continuum. Unmotivated students are the most likely to feel negative emotions (Ratelle et al., 2007; David, 2010), to have low self-esteem (Deci and Ryan, 1995), and who attempts to abandon their studies (Vallerand et al., 1997; Blanchard et al., 2005). So, more students are motivated by external regulation or demotivated, less interest they show and less effort they make, and more likely they are to fail (Grolnick et al., 1991; Miserandino, 1996; Guay et al., 2000; Blanchard et al., 2005).

It is worth noting that there is a close link between motivation and learning (Bessa et al., 2021; Rossa et al., 2021). Indeed, when the learner's motivation is high, so will his learning. However, all this depends on the method used (Norboev, 2021). For example, the method of problem-solving increase motivation more than the traditional method, as evidenced by several researchers (Parish and Treasure, 2003; Artino and Stephens, 2009; Kim and Frick, 2011; Lemos and Veríssimo, 2014).

Given the effectiveness of the problem-solving method in improving students' learning and motivation, it should be used during physical education teaching. This could be achieved through the organization of comprehensive training programs, seminars, and workshops for teachers so to master and subsequently be able to use the problem-solving method during physical education lessons.

Despite its novelty, the present study suffers from a few limitations that should be acknowledged. First, a future study, consisting of a group taught using the mixed method would preferable so to better elucidate the true impact of this teaching and learning method. Second, no gender and/or age group comparisons were performed. This issue should be addressed in future investigations. Finally, the number of participants is limited. This may be due to working in a secondary school where the number of students in a class is limited to 30 students. Additionally, the number of participants fell to 53 after excluding certain students (exempted, absent for a session, exercising in civil clubs or member of the school association). Therefore, to account for classes of finite size, a cluster-based trial would be beneficial in the future. Moreover, future studies investigating the effect of the active method in reducing some behaviors (e.g., disruptive behaviors) and for the improvement of pupils' attention are warranted.

## 5. Conclusion

There was an improvement in student learning in favor of the problem-solving method. Additionally, we found that the motivation of learners who were taught using the problem-solving method was better than that of learners who were educated by the traditional method.

## Data availability statement

The original contributions presented in the study are included in the article/supplementary material, further inquiries can be directed to the corresponding author.

## Ethics statement

University Research Ethics Board approval was obtained before recruiting participants who were subsequently informed of the nature, objective, methodology, and constraints. Teacher, school director, parental/guardian, and child informed consent was obtained prior to participation in the study. In addition, exclusion criteria included; the practice of handball activity in civil/competitive/amateur clubs or in the high school sports association. Written informed consent to participate in this study was provided by the participants' legal guardian/next of kin.

## Author contributions

All authors listed have made a substantial, direct, and intellectual contribution to the work and approved it for publication.
